# Whole blood-derived microRNA signatures in mice exposed to lipopolysaccharides

**DOI:** 10.1186/1423-0127-19-69

**Published:** 2012-07-31

**Authors:** Ching-Hua Hsieh, Cheng-Shyuan Rau, Jonathan Chris Jeng, Yi-Chun Chen, Tsu-Hsiang Lu, Chia-Jung Wu, Yi-Chan Wu, Siou-Ling Tzeng, Johnson Chia-Shen Yang

**Affiliations:** 1 Department of Plastic and Reconstructive Surgery, Kaohsiung Chang Gung Memorial Hospital, Chang Gung University College of Medicine, Kaohsiung, Taiwan; 2 Department of Neurosurgery, Kaohsiung Chang Gung Memorial Hospital and Chang Gung University College of Medicine, Kaohsiung, Taiwan; 3 Business BA at University of Texas at Dallas, 800 W Campbell Road, Richardson, TX, 75080, USA

**Keywords:** MicroRNAs, Lipopolysaccharide, Lipoteichoic acid, Toll-like receptor, Gram-negative bacteria, Gram-positive bacteria, Microarray

## Abstract

**Background:**

Lipopolysaccharide (LPS) is recognized as the most potent microbial mediator presaging the threat of invasion of Gram-negative bacteria that implicated in the pathogenesis of sepsis and septic shock. This study was designed to examine the microRNA (miRNA) expression in whole blood from mice injected with intraperitoneal LPS.

**Methods:**

C57BL/6 mice received intraperitoneal injections of varying concentrations (range, 10–1000 μg) of LPS from different bacteria, including *Escherichia coli*, *Klebsiella pneumonia*, *Pseudomonas aeruginosa*, *Salmonella enterica*, and *Serratia marcescens* and were killed 2, 6, 24, and 72 h after LPS injection. Whole blood samples were obtained and tissues, including lung, brain, liver, and spleen, were harvested for miRNA expression analysis using an miRNA array (Phalanx miRNA OneArray® 1.0). Upregulated expression of miRNA targets in the whole blood of C57BL/6 and *Tlr4*^*−/−*^ mice injected with LPS was quantified using real-time RT-PCR and compared with that in the whole blood of C57BL/6 mice injected with lipoteichoic acid (LTA) from *Staphylococcus aureus*.

**Results:**

Following LPS injection, a significant increase of 15 miRNAs was observed in the whole blood. Among them, only 3 miRNAs showed up-regulated expression in the lung, but no miRNAs showed a high expression level in the other examined tissues. Upregulated expression of the miRNA targets (let-7d, miR-15b, miR-16, miR-25, miR-92a, miR-103, miR-107 and miR-451) following LPS injection on real-time RT-PCR was dose- and time-dependent. miRNA induction occurred after 2 h and persisted for at least 6 h. Exposure to LPS from different bacteria did not induce significantly different expression of these miRNA targets. Additionally, significantly lower expression levels of let-7d, miR-25, miR-92a, miR-103, and miR-107 were observed in whole blood of *Tlr4*^−/−^ mice. In contrast, LTA exposure induced moderate expression of miR-451 but not of the other 7 miRNA targets.

**Conclusions:**

We identified a specific whole blood–derived miRNA signature in mice exposed to LPS, but not to LTA, from different gram-negative bacteria. These whole blood-derived miRNAs are promising as biomarkers for LPS exposure.

## Background

Lipopolysaccharide (LPS) from gram-negative bacteria is a potent inflammatory stimulus and is often administered as an infectious insult. LPS-induced Toll-like receptor 4 (TLR4) signal transduction activates well-characterized pathways, including those involving nuclear factor-kappa B (NF-κB) and activator protein 1 (AP-1), leading to the production of downstream pro-inflammatory cytokines, chemokines, or leukocyte adhesion molecules [1,2]. When an initial host defense to an infection magnifies, sepsis can develop, leading to severe adverse outcomes such as organ dysfunction and death [3]; therefore, early diagnosis of bacterial infection is clinically important.

MicroRNAs (miRNAs) are approximately 22-nt–long small regulatory RNA molecules that modulate the activity of specific mRNA targets and play important roles in a wide range of physiologic and pathologic processes [4,5]. Alteration of miRNA expression profiles has been observed in various diseases, including cancer, cardiovascular diseases, neurological diseases, and several inflammatory and autoimmune diseases. Further, differential expression of miRNAs may help distinguish between disease states [6]. miRNA expression regulated by LPS target genes reportedly contributes to inflammatory phenotypes [7]. Furthermore, some studies have identified miRNAs as important regulators of immune responses [8-10] and as fine-tuners of Toll-like receptors (TLRs) [11-13]. Although ribonuclease is present in both plasma and serum, extracellular miRNAs circulate in the blood of both healthy and diseased patients and are remarkably stable, making their isolation and analysis easy [14]. Biochemical analyses indicate that miRNAs are resistant to RNase activity, extreme pH and temperature, extended storage, and large numbers of free-thaw cycles [15,16]. In contrast to mRNAs, miRNAs are themselves active moieties and should thus reflect physiological alterations more directly [17]. Compared to protein-based blood biomarkers, most circulating miRNAs can be easily detected by PCR, and low abundance can significantly hinder the detection of some protein-based biomarkers [18]. Additionally, protein-based biomarkers may have different post-translational modifications that can affect the accuracy of measurement, while miRNAs are relatively homogeneous [18]. Moreover, with the possibility to analyze multiple miRNAs in parallel to increase sensitivity and specificity by using complex miRNA expression patterns, miRNAs might constitute very useful and accessible diagnostic tools in a cluster pattern [15,17]. The present study was designed to profile miRNA expression levels in whole blood during *in vivo* exposure to LPS in mice.

## Methods

### Animal experiments

C57BL/6 mice were purchased from BioLasco (Taiwan). *Tlr4*^*−/−*^ (C57BL/10ScNJ) mice were purchased from Jackson Laboratory (Bar Harbor, ME, USA). The mice were maintained in a pathogen-free environment and had access to food and water *ad libitum*. LPS from different bacteria, including *Escherichia coli* serotype 026:B6 (catalog no. L3755), *Klebsiella pneumonia* (L1519), *Pseudomonas aeruginosa* (L9143), *Salmonella enterica* serotype Enteritidis (L6761), and *Serratia marcescens* (L6136) were purchased from Sigma (St. Louis, MO, USA). When the mice gained a weight of 20–35 g and became 4–6 weeks old, they were intraperitoneally injected with 10, 100, 1000 μg of LPS reconstituted in 100 μL of phosphate-buffered saline (PBS). Animals were sacrificed at 2, 6, 24, and 72 h after LPS injection. The control group was injected with 100 μL PBS. Whole blood was drawn and tissues, including lung, brain, liver and spleen, were harvested for miRNA expression analysis. For comparison, intraperitoneal injections of 10, 100, 1000 μg of lipoteichoic acid (LTA) from *Staphylococcus aureus* (L2515, Sigma) were performed; animals were killed 6 h after injection, and whole blood was drawn. All housing conditions were established and surgical procedures, analgesia, and assessments were performed according to the Animal Care Guidelines and protocols approved by the Animal Care Committee at Chang Gung Memorial Hospital.

### RNA isolation and preparation

For miRNA detection, whole blood samples (1 mL per mouse) were collected into tubes containing EDTA. Total RNA was extracted from whole blood and harvested tissue by using the RNeasy Mini kit (Qiagen, Hilden, Germany). Purified RNA was quantified by measuring the absorbance at 260 nm by using an SSP-3000 Nanodrop spectrophotometer (Infinigen Biotechnology, Inc., City of Industry, CA, USA). For miRNA array analyses, the quality of purified RNA was assessed using a Bioanalyzer 2100 (Agilent Technologies, Santa Clara, CA, USA). Total RNA (2 μg) was reverse transcribed into cDNA by using the TaqMan miRNA Reverse Transcription Kit (Applied Biosystems, Foster City, CA, USA). Target miRNA was reverse transcribed using sequence-specific stem-loop primers. miRNA cDNA (10 ng) for each target was used for real-time PCR.

### miRNA microarray analysis

The Mouse & Rat miRNA OneArray® 1.0 (Phalanx Biotech Group, Hsinchu, Taiwan) contains a total of 2,319 probes, including 135 experimental control probes and 728 unique miRNA probes from mouse (miRBase Release 12.0) and 348 from rat (miRBase Release 12.0). Mouse genome-wide miRNA microarray analysis was performed by Phalanx Biotech. Briefly, fluorescent targets were prepared from 2.5-μg total RNA samples by using the miRNA ULS^TM^ Labeling Kit (Kreatech Diagnostics, Amsterdam, Netherlands). Labeled miRNA targets enriched using NanoSep 100K (Pall Corporation, Port Washington, NY, USA) were hybridized to the Mouse & Rat miRNA OneArray® 1.0 with Phalanx hybridization buffer by using the OneArray® Hybridization Chamber. After overnight hybridization at 37°C, non-specific binding targets were by 3 washing steps (Wash I: 37°C, 5 min; Wash II: 37°C, 5 min and 25°C, 5 min; and Wash III: rinse 20 times). The slides were dried by centrifugation and scanned using Axon 4000B scanner (Molecular Devices, Sunnyvale, CA, USA). The Cy5 fluorescent intensities of each spot were analyzed using GenePix 4.1 software (Molecular Devices). The signal intensity of each spot was processed using the R program. We filtered out spots for which the flag was <0. Spots that passed the criteria were normalized using the 75% media scaling normalization method. Normalized spot intensities were converted into gene expression log_2_ ratios for the control and treatment groups. Spots with log_2_ ratios ≥ 1 or log_2_ ratio ≤ −1 and *P*-value < 0.05 are analyzed further. These differentially expressed miRNAs were subjected to hierarchical cluster analysis using average linkage and Pearson correlation as a measure of similarity. The GEO accession number for the microarray data is GSE36472.

### Quantification of miRNA expression

miRNA expression was quantified by real-time RT-PCR using Applied Biosystems 7500 Real*-*Time PCR System (Applied Biosystems) to verify the miRNA targets with up-regulated expression that were detected through the miRNA array from whole blood following injection of LPS different doses (10, 100, and 1000 μg) or at indicated survival times (2, 6, 24, and 72 h). Expression of the miRNAs in whole blood following intraperitoneal injections of LTA at doses of 10, 100, and 1000 μg were measured for comparison. Expression of each miRNA was represented relative to the expression of U6 small nuclear RNA (*RUN6B*) as an internal control. We calculated the fold-expression of induction as the relative expression value obtained from 6 samples in comparison with that from the control group. Intergroup group comparisons were performed using analysis of variance (ANOVA) and an appropriate post hoc test to compensate for multiple comparisons (SigmaStat; Jandel, San Rafael, CA, USA). *P*-values < 0.05 were considered significant.

## Results

### Up-regulated miRNA targets in microarray analysis

**Figure 1:**
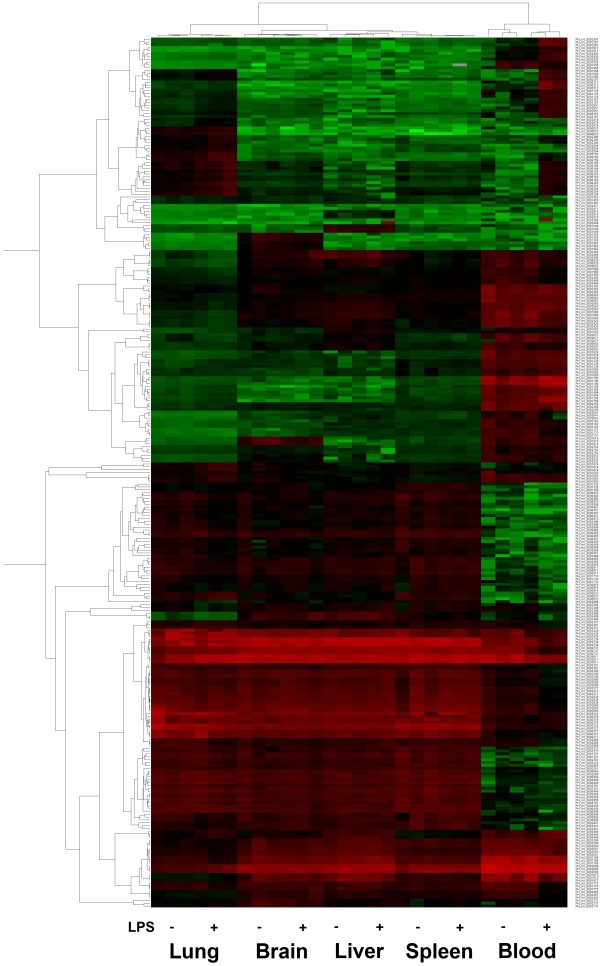
**Hierarchical cluster analysis of significant expression of miRNA in lung, brain, liver, spleen, and whole blood of C567BL/6 mice 6 h after 100-μg LPS injection.** The indicated miRNA name by the corresponding gene probe in Phalanx array could be found in: http://www.phalanx.com.tw/Products/MRmiOA_Probe.php.

Expression of miRNA was considered significantly different when values for all 5 samples from the whole blood, lung, brain, liver, and spleen of experimental mice at 6 h after 100 μg LPS (L3755) injections were more than double of those for the controls (n = 3 for each subgroup). The hierarchical cluster analysis of all significant miRNAs is shown in Figure 1, which illustrates miRNAs differentially expressed in different tissues. Unsupervised hierarchy clustering was used to separate different sample tissues into different groups. The up-regulated miRNA targets more than double of those of the control is shown in Table 1. 15 miRNAs (miR-223, miR-155, miR-21, miR-21*, miR-667, let-7a, miR-146a, miR-107, miR-34b-5p, miR-146b, miR-133a, miR-15a, miR-191, miR-103, and miR-16) showed significantly up-regulated expression in the lung tissue. No miRNA showed upregulated expression in the brain. Only miR-292-5p and miR-155, miR-21*, and miR-101a showed upregulated expression in the liver and spleen, respectively. There were 15 miRNAs showed significantly increased expression in the whole blood. Among the miRNAs with upregulated expression levels in the whole blood, only 4—miR-16, miR-103, miR-107, and let-7a—showed high expression in the lung. However, no miRNA with upregulated expression levels in the whole blood showed high expression levels in other tissues, including the brain, liver, or spleen. The down-regulated miRNA targets more than double of those of the control is shown in Table 2. There were 23, 10, 8, and 51 miRNAs showed significantly down-regulated expression in the lung, liver, spleen and blood sample, respectively. No miRNA showed upregulated expression in the brain. Among the miRNAs with down-regulated expression levels in the whole blood, miR-690, miR-34b-3p, and miR-34b-3p also showed low expression in the liver, lung, and spleen, respectively. Besides, miR-466d-5p showed low expression in both lung and spleen. Most of the down-regulated miRNAs in the whole blood did not have similar low expression levels in the tissues of lung, brain, liver, and spleen.

**Table 1:** Up-regulated miRNA targets (more than double of those of the control) in samples from lung, brain, liver, spleen, and whole blood of experimental mice 6 h following 100-μg LPS (L3755) injection

**Lung**	**Fold (log**_**2**_**)**	**Brain**	**Fold (log**_**2**_**)**	**Liver**	**Fold (log**_**2**_**)**	**Spleen**	**Fold (log**_**2**_**)**	**Blood**	**Fold (log**_**2**_**)**
miR-223	2.65	nil		miR-292-5p	1.07	miR-155	1.76	miR-15b	3.65
miR-155	2.07					miR-21*	1.60	miR-451	3.63
miR-21	1.38					miR-101a	1.03	miR-16	3.43
miR-21*	1.32							miR-103	2.74
miR-667	1.20							miR-107	2.28
let-7a	1.18							let-7d	2.23
miR-146a	1.12							miR-92a	2.07
miR-107	1.12							miR-25	2.05
miR-34b-5p	1.08							let-7b	1.98
miR-146b	1.07							let-7a	1.97
miR-133a	1.07							let-7c	1.80
miR-15a	1.04							let-7i	1.69
miR-191	1.03							miR-652	1.62
miR-103	1.02							miR-92b	1.60
miR-16	1.01							miR-590-5p	1.17

*miRNA* microRNA; *LPS* lipopolysaccharide.

**Table 2:** Down-regulated miRNA targets (more than double of those of the control) in samples from lung, brain, liver, spleen, and whole blood of experimental mice 6 h following 100-μg LPS (L3755) injection

**Lung**	**Fold (log**_**2**_**)**	**Brain**	**Fold (log**_**2**_**)**	**Liver**	**Fold (log**_**2**_**)**	**Spleen**	**Fold (log**_**2**_**)**	**Blood**	**Fold (log**_**2**_**)**
miR-698	−2.12	nil		miR-130a	−2.92	miR-468	−1.35	miR-1195	−3.05
miR-467b*	−1.78			miR-126-3p	−2.75	miR-672	−1.27	miR-667	−2.38
miR-466b-3-3p	−1.69			miR-193	−2.55	miR-214*	−1.21	miR-690	−2.31
miR-376a	−1.61			miR-690	−2.06	miR-466d-5p	−1.17	miR-880	−2.30
miR-211	−1.53			miR-805	−1.92	miR-466b-5p	−1.10	miR-34b-3p	−2.10
miR-466a-3p	−1.50			miR-221	−1.34	miR-18b	−1.08	miR-709	−1.96
miR-471	−1.49			miR-29b	−1.33	miR-16*	−1.04	miR-468	−1.79
miR-875-3p	−1.46			miR-146a	−1.27	miR-669e	−1.00	miR-1186	−1.68
miR-493	−1.45			miR-17	−1.21			miR-710	−1.67
miR-694	−1.41			miR-22	−1.16			miR-1897-5p	−1.65
miR-704	−1.39							miR-466j	−1.63
miR-574-3p	−1.26							miR-155	−1.59
miR-485*	−1.25							miR-294*	−1.58
miR-470	−1.23							miR-1898	−1.55
miR-466d-5p	−1.21							miR-380-3p	−1.54
let-7a*	−1.19							miR-703	−1.52
miR-34b-3p	−1.17							miR-342-3p	−1.51
miR-293	−1.14							miR-466d-5p	−1.51
miR-1902	−1.13							miR-206	−1.48
miR-494	−1.09							miR-678	−1.47
miR-467f	−1.08							miR-691	−1.44
miR-204	−1.07							miR-532-5p	−1.44
miR-294	−1.06							miR-685	−1.43
								miR-1188	−1.42
								miR-466f-5p	−1.38
								miR-483	−1.37
								miR-326	−1.37
								miR-380-5p	−1.36
								miR-495	−1.35
								miR-680	−1.33
								miR-141*	−1.32
								miR-467e*	−1.32
								miR-1896	−1.32
								miR-193b	−1.31
								miR-99b*	−1.31
								miR-673-5p	−1.30
								miR-882	−1.30
								miR-1895	−1.27
								miR-133b	−1.26
								miR-491	−1.26
								miR-214	−1.24
								miR-339-5p	−1.23
								miR-467h	−1.23
								miR-134	−1.22
								miR-466h	−1.21
								miR-370	−1.20
								miR-878-5p	−1.20
								miR-196a*	−1.20
								miR-150	−1.20
								miR-133a	−1.18
								miR-186	−1.17

*miRNA* microRNA; *LPS* lipopolysaccharide.

### Expression profiles of miRNAs

**Figure 2:**
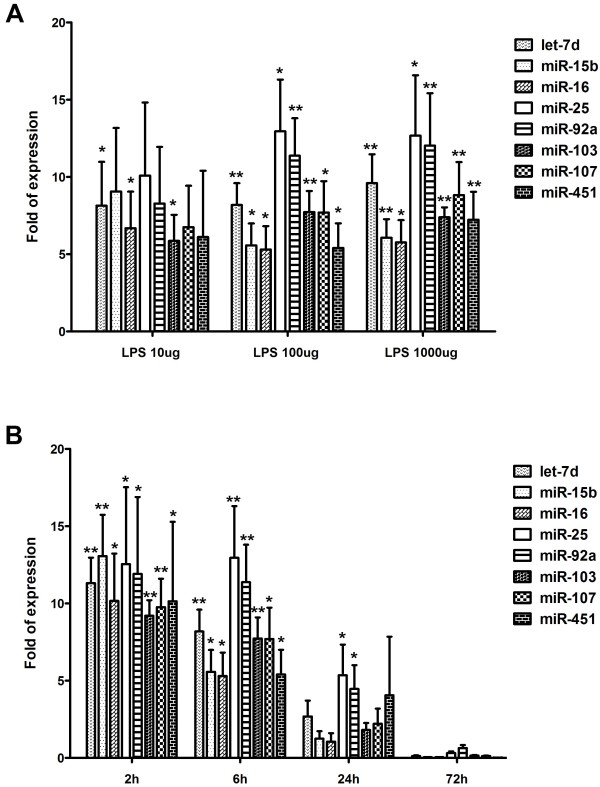
**(A) Dose- and (B) time-dependent upregulation of miRNA expression.** Expression of representative up-regulated miRNAs (let-7d, miR-15b, miR-16, miR-25, miR-92a, miR-103, miR-107, and miR-451) identified using miRNA microarray of whole blood using real-time RT-PCR. Whole blood was drawn following injection of 10, 100, 1000 μg LPS; mice were killed at the indicated survival times (2, 6, 24, and 72 h). Bars represent means ± SEM of 6 experiments; *, *P* < 0.05 vs. control; **, *P* < 0.01 vs. control.

**Figure 3:**
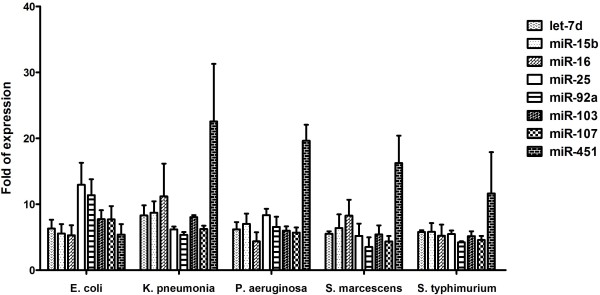
**Expression of let-7d, miR-15b, miR-16, miR-25, miR-92a, miR-103, miR-107, and miR-451 from whole blood of C57BL/6 mice based on real-time RT-PCR experiments 6 h after exposure to 100 μg LPS originating from different bacteria, including*Escherichia coli*serotype 026:B6,*Klebsiella pneumonia*,*Pseudomonas aeruginosa*,*Salmonella enterica*, serotype Enteritidis, and*Serratia marcescens.***

Considering the signaling pathway which induces the miRNA transcription directly upon LPS stimulation and suitable number of miRNA targets for biomarkers, expression of miRNAs in whole blood following LPS injection was quantified using real-time RT-PCR to verify selected up-regulated, but not down-regulated, miRNA targets with at least 4-fold increase in expression (let-7d, miR-15b, miR-16, miR-25, miR-92a, miR-103, miR-107, and miR-451). The above-mentioned 8 miRNAs showed approximately 5- to 12-fold increase in expression 6 h after 100 and 1000 μg LPS (L3755) injection (Figure 2A). Following injection with 10 μg of LPS, increased expression was observed in the 8 miRNAs, but expression levels of only let-7d, miR-16, and miR-103 were significantly higher than the control (Figure 2A). In contrast, upon 100 ug and 1000 ug of LPS injection, all these 8 miRNAs (let-7d, miR-15b, miR-16, miR-25, miR-92a, miR-103, miR-107, and miR-451) had a significant expression than the control (Figure 2A). Therefore, 100 ug of LPS was chosen for injection in the subsequent experiment and we found the induction of the above-mentioned 8 miRNAs was evident as early as 2 h and persisted for at least 6 h following injection with 100 ug of LPS (Figure 2B). At 24 h, only miR-25 and miR-92a continued to be significantly expressed in the blood. No up-regulation of these 8 miRNA targets was detected 72 h after LPS injection. Additionally, no significant difference was observed between groups 6 h after exposure to 100 μg LPS (Figure 3).

### miRNA expression in Tlr4 knockout mice

**Figure 4:**
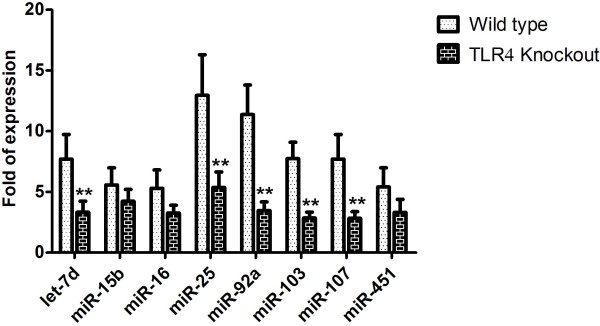
**Expression of let-7d, miR-15b, miR-16, miR-25, miR-92a, miR-103, miR-107, and miR-451 of whole blood from C57BL/6 and*Tlr4***^***−/−***^(C57BL/10ScNJ) mice from real-time RT-PCR experiments 6 h after exposure to 100 μg LPS; **,*P* **< 0.01 vs. control.**

To investigate the role of the TLR4 receptor in inducing expression of the miRNA targets, expression of let-7d, miR-15b, miR-16, miR-25, miR-92a, miR-103, miR-107, and miR-451 in the whole blood of *Tlr4*^−/−^ mice 6 h after intraperitoneal injection of 100 μg LPS (L3755) was measured against that from the whole blood of *Tlr4*^−/−^ mice injected with PBS. In TLR4 receptor knockout mice, significantly lower expression of let-7d, miR-25, miR-92a, miR-103 and miR-107 was observed following LPS treatment (Figure 4). The expression of these 8 miRNAs was not significantly different between control C57BL/6 and *Tlr4*^−/−^ mice.

### miRNA expression after LTA injection

**Figure 5:**
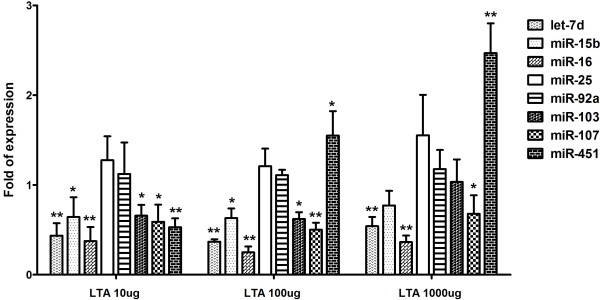
Expression of let-7d, miR-15b, miR-16, miR-25, miR-92a, miR-103, miR-107, and miR-451 of whole blood from C57BL/6 mice using real-time RT-PCR experiment 6 h after exposure to 10, 100, and 1000 μg LTA originating from*Staphylococcus aureus*; *,*P* < 0.05 vs. control; **,*P* **< 0.01 vs. control.**

To investigate that whether lipoteichoic acid (LTA) originating from gram-positive bacteria induces expression of let-7d, miR-15b, miR-16, miR-25, miR-92a, miR-103, miR-107, and miR-451, whole blood was drawn at 6 h following intraperitoneal injections of 10, 100, or 1000 μg LTA from *S. aureus* for real-time PCR. The results showed that LTA only moderately induced miR-451 expression at concentrations of 100 and 1000 μg. Notably, LTA did not up-regulate the expression of the other 7 miRNA targets, and decreased the expression levels of let-7d, miR-15b, miR-16, miR-103, and miR-107 at the various concentrations tested (Figure 5).

## Discussion

In humans and other mammals, innate immunity generally preserves host integrity with respect to the molecular components of invading microbial pathogens through pattern recognition receptors (PRRs). PRRs are germ line-encoded receptors that sense specific pathogen-associated molecular patterns (PAMPs), such as LPS, and activate responses in cells against the pathogens. While TLR4 recognizes LPS, TLR2, in association with TLR1 or TLR6, recognizes LTA originating from gram-positive bacteria [19]. LPS and LTA induce similar inflammatory responses, and their activation results in either symptoms of sepsis or shock. However, while there are similarities between these responses, signaling and sensing of LPS and LTA differ significantly. Bacterial LPS consists of a hydrophobic lipid A domain, an oligosaccharide core, and a distal polysaccharide (the O antigen) [20]. The lipid A moiety alone is sufficient for activating the innate immune response; adaptive (antibody) responses are generated against the O antigen polysaccharide later during infection [1]. Lipid A consists of a diglucosamine diphosphate head group substituted with 4–8 acyl chains in different bacterial species. Nevertheless, many bacteria produce lipid A species that are similar to *E. coli* lipid A, which contains a diglucosamine diphosphate head group and 6 acyl chains, and are powerful immunostimulants [1]. In this study, we demonstrated that expression of multiple miRNAs (let-7d, miR-15b, miR-16, miR-25, miR-92a, miR-103, miR-107, and miR-451) is significantly altered in the whole blood of mice after exposure to LPS in a dose- and time-dependent fashion. LPS from different gram-negative bacteria, including *E. coliK. pneumoniaP. aeruginosaS. enterica*, and *S. marcescens,* induced similar up-regulation of all the miRNAs examined. miRNA profiles from whole blood may be detectable during very early stages following exposure to LPS, including as early as 2 h after treatment. Additionally, miRNA targets were not up-regulated following treatment with LTA. Thus, this miRNAs expression signature may be useful in differentiating infections caused by gram-negative and gram-positive bacteria.

Despite accumulating evidence of miRNAs in the blood and body fluids, the origin, and particularly, the function of these circulating extracellular miRNAs remains poorly understood [21]. Most circulating miRNAs are part of larger lipid or lipoprotein complexes, such as apoptotic bodies, microvesicles, or exosomes, and are highly stable [15,17,21]. Currently, little is known regarding the biologic roles of these molecules at distant sites in the body [22]. Extracellular miRNAs may be mediators of cell–cell communication [23,24]. Previous studies have shown that circulating miRNAs in body fluids and extracellular fluid compartments show hormone-like effects, leading to widespread responses within cells at some distance away from the secreting cell [25]. Expression of circulating miRNAs is thought to reflect extrusion of miRNAs from relevant remote tissues or organs or disease processes [16]. However, it is likely that peripheral blood miRNAs do not only reflect miRNAs expressed in remote tissues [26]. Studies have also demonstrated that nearly 30% of the released miRNAs *in vitro* and *in vivo* do not reflect the expression profile found in donor cells, suggesting that specific miRNAs are selected to be intracellularly retained or released by exosomes [27]. Indeed, the result of this study showed that miRNAs are differentially expressed in blood and various tissues. Only 3 miRNAs with upregulated expression (miR-16, miR-103, and miR-107) in whole blood showed high expression in the lung, and no miRNA in whole blood showed high expression in other tissues, including brain, liver, or spleen. Additionally, following exposure to LPS, expression of only 5 miRNAs (let-7d, miR-25, miR-92a, miR-103, and miR-107) was significantly lower in TLR4 receptor knockout mice. Among them, only miR-107 had been reported to be associated with TLR4 upon LPS stimulation in the literature [28]. Let-7 family has been shown to function as a tumor suppressor through regulating multiple oncogenic signaling [29]. Knockdown of let-7d promote epithelial-mesenchymal transition (EMT) traits and migratory/invasive capabilities in oral squamous cell carcinoma cells [29]. miR-25 were significantly upregulated in the serum of patients with hepatitis B virus (HBV)-positive hepatocellular carcinoma [30]. MiR-92a appears to target mRNAs corresponding to several proangiogenic proteins and controls angiogenesis and functional recovery of ischemic tissues [31]. Overexpression of miR-92a decreased the expression of the transcription factor Krüppel-like factor 2 (KLF2), which is crucial for maintaining endothelial function, and the KLF2-regulated endothelial nitric oxide synthase and thrombomodulin [31]. In a maternal and fetal liver of hepatitis B virus (HBV) transgenic mouse model, expression of miR-92a increased by more than 6-fold in the fetal livers and implicated in HBV intrauterine infection [32]. The miR-103 and miR-107 in the circulation have a crucial role in regulating insulin and glucose homeostasis [33]. High expression of miR-103 and miR-107 (miR-103/107) was found in the presence of hypoxia, thereby potentiating metastasis suppressors death-associated protein kinase (DAPK) and Krüppel-like factor 4 (KLF4) downregulation and hypoxia-induced motility and invasiveness of colorectal cancer cell lines [34]. Serum miR-103 can also serve as a potential diagnostic marker for breast cancer [35]. Recently, TLR4 has been shown to increase cyclin-dependent kinase 6 (CDK6) expression by down-regulating miR-107 in macrophages [28], that observation seemed to be different from our study showing an up-regulated miR-107 via TLR4 upon LPS treatment. We speculated that the expression of miR-107 may be contributed by other cells. However, the source of circulating miRNAs is far from understood and yet to be clarified. In addition, following exposure to LPS, expression of 3 miRNAs (miR-15b, miR-16, and miR-451) was not significantly lower in TLR4 receptor knockout mice. Notably, the levels of miR-16 and miR-451, both present in significant levels in red blood cells, were the major source of variation in miR-16 and miR-451 levels measured in the circulation [36]. Although a few articles have suggested a TLR4-independent upon LPS stimulation in the interleukin 1 (IL-1) signaling [37,38], given the multiple systemic responses following *in vivo* administration of LPS, so far it is unclear whether this observed up-regulation of miRNA expression is the direct result of TLR4 signaling or events secondary to systemic TLR4 activation. Whether there is a direct TLR4-independent signal pathway in the induction of miRNA expression upon LPS stimulation required further investigation and validation.

Normalization of circulating miRNAs is critical for objectively evaluating their expression levels; however, there are currently no known standard extracellular housekeeping miRNAs that can be used for normalization [21]. Although previous studies have reported the use of miR-16 and miR-142-3p, which show relatively stable expression in the serum, as endogenous controls [39,40], it is unknown whether they are stable in the circulation. In our study, miR-16 expression was upregulated by approximately 5-fold following LPS treatment, preventing its use as an internal control. Genes typically used for reference, such as *RNU6B* and 5S ribosomal RNA, were found to have less stable expression in serum samples. In contrast, expression of whole blood–derived miRNAs was more stable and consistent. Profiles of miRNAs from whole blood have been examined previously [41-43]. Addition of synthetic miRNAs from other organisms such as *Caenorhabditis elegans* in serum may be a useful approach for endogenous control during qRT-PCR. However, additional studies are necessary to develop an accurate normalization protocol and empirical validation method for stable endogenous control of miRNAs for each type of body fluid [25].

Early diagnosis of bacterial infection is critical for preventing further complications. Although microbiological culture is the standard method for identifying infective bacterial species, this technique is time-consuming, which can delay treatment. Potential biomarkers include acute phase protein, cytokines, and chemokines, which are not sufficiently specific to differentiate between gram-negative and gram-positive bacterial infection, which is compounded by overlap with other inflammatory diseases [44]. We identified whole blood–derived miRNAs following LPS exposure as promising biomarkers; however, future studies should be performed to clarify the origin and physiological role of circulating miRNAs. Further investigation is required to understand the expression of miRNAs in gram-negative or gram-positive bacterial infection. Additionally, studies examining miRNA expression levels at different stages of infection and intervention using antibiotic treatments are also necessary.

## Conclusion

We identified a specific whole blood–derived miRNA signature in mice exposed to LPS, but not to LTA, from different gram-negative bacteria.With a dose- and time-dependentupregulated expression of the miRNA targets (let-7d, miR-15b, miR-16, miR-25, miR-92a, miR-103, miR-107 and miR-451) following LPS injection, these whole blood-derived miRNAs are promising as biomarkers for LPS exposure.

## Competing interests

The authors declare no potential conflict of interests.

## Authors’ contributions

CHH was responsible for the writing of the manuscript. CSR participated in the analysis and interpretation of the data.JCJ and THL participated in the animal surgery and acquisition of the study specimens. YCC and CJW participated in the real-time RT-PCR experiment. YCWwere involved in the acquisition of the miRNA array and whole genome expression data.SLTparticipated by providing and coordinating the resources.YCCcontributed tothe design and coordination of the data acquisition and analysis. All authors read and approved the final manuscript.
